# 

**Published:** 1877-05

**Authors:** 


*CONTENTS.*

| | PAGE |
|---|---|
| THE GRAVE OF HOPE | 121 |
| CONSTIPATION | 124 |
| THE IRISH | 124 |
| SPRING DISEASES | 125 |
| GRATUITOUS MEDICAL ADVICE | 126 |
| CHILDREN EATING | 126 |
| TEMPERAMENT DIFFERENCES | 127 |
| WELL DONE | 129 |
| BATHS AND BATHING | 129 |
| SUDDEN DEATH | 130 |
| QUENCHING THIRST | 132 |
| DRINKING AND DEATH | 132 |
| LIQUOR DRINKING | 133 |
| DANGERS OF SPRING | 134 |
| PURE FOOD | 135 |
| SECOND CILDHOOD | 136 |
| BULL DOGS | 136 |
| SORROWING POVERTY | 139 |
| WEAKLY YOUTHS | 140 |
| WALK SOFTLY | 140 |
| BEAUTIFUL OLD AGE | 141 |
| LIVING AGES | 142 |
| OBJECT OF EATING | 143 |
| REFORMING A REFORMER | 144 |
| WHEAT GLUTEN | 146 |
| A GRAND LECTURE | 147 |
| SPRING AILMENTS | 148 |
| THE EYES | 148 |
| IMPROVING TIME | 149 |
| BOUND VOLUMES | 149 |

